# Development of a novel gut microphysiological system that facilitates assessment of drug absorption kinetics in gut

**DOI:** 10.1038/s41598-024-80946-6

**Published:** 2024-12-02

**Authors:** Tomoki Imaoka, Reiko Onuki-Nagasaki, Hiroshi Kimura, Kempei Tai, Mitsuharu Ishii, Ayaka Nozue, Ikuko Kaisaki, Misa Hoshi, Kengo Watanabe, Kazuya Maeda, Takashi Kamizono, Takahiro Yoshioka, Takashi Fujimoto, Taku Satoh, Hiroko Nakamura, Osamu Ando, Hiroyuki Kusuhara, Yuzuru Ito

**Affiliations:** 1 Drug Metabolism & Pharmacokinetics Research Laboratories, Daiichi Sankyo Co., Ltd, 1-2-58, Hiromachi, Shinagawa-ku, Tokyo, 140-8710 Japan; 2 Institute of Life and Environmental Sciences, University of Tsukuba, 1-1-1 Tennodai, Tsukuba, 305-8572 Ibaraki Japan; 3 Micro/Nano Technology Center, Tokai University, 4-1-1 Kitakaname, Hiratsuka, 259-1292 Kanagawa Japan; 4 Graduate School of Pharmaceutical Sciences, The University of Tokyo, 7-3-1 Hongo, Bunkyo- ku, Tokyo, 113-0033 Japan; 5 Kitasato University School of Pharmacy, 5-9-1 Shirokane, Minato-ku, Tokyo, 108-8641 Japan; 6 Tokyo Ohka Kogyo Co. Ltd, Samukawa-machi, Koza-gun, Tabata, 1590, 253-0114 Kanagawa Japan

**Keywords:** Drug discovery, Pharmaceutics

## Abstract

There is an urgent need for novel methods that can accurately predict intestinal absorption of orally administered drugs in humans. This study aimed to evaluate the potential of a novel gut microphysiological system (MPS), gut MPS/Fluid3D-X, to assess the intestinal absorption of drugs in humans. The gut MPS/Fluid3D-X model was constructed using a newly developed flow-controllable and dimethylpolysiloxane-free MPS device (Fluid3D-X^®^). Human induced pluripotent stem cells-derived small intestinal epithelial cells were employed in this model, which exhibited key characteristics of the human absorptive epithelial cells of the small intestine, including the expression of key gene transcripts responsible for drug transport and metabolism, and the presence of dome-like protrusions in the primary intestinal epithelium under air-liquid interface culture conditions. Functional studies of transporters in the constructed model demonstrated basal-to-apical directional transport of sulfasalazine and quinidine, substrates of the active efflux transporters breast cancer resistance protein and P-glycoprotein, respectively, which were diminished by inhibitors. Furthermore, a cytochrome P450 (CYP) 3A inhibitor increased the apical-to-basal transport of midazolam, a typical CYP3A4 substrate, and reduced metabolite formation. These results suggest that gut MPS/Fluid3D-X has the potential to assess the intestinal absorption of small-molecule drugs.

**Supplementary Information:**

The online version contains supplementary material available at 10.1038/s41598-024-80946-6.

## Introduction

The intestine plays a crucial role in determining the systemic exposure of orally administered drugs. Intestinal absorption involves multiple processes including permeability across the intestinal epithelial barrier, escape the barrier function of efflux transporters such as P-glycoprotein (P-gp) and breast cancer resistance protein (BCRP) and intestinal metabolism including cytochrome P450 or UDP-glucuronosyltransferases^1–3^. Based on our understanding of the key factor(s) governing intestinal absorption, predicting the net effect of these factors is essential for selecting drugs with favorable intestinal absorption properties in humans. During drug discovery and development, intestinal absorption is commonly studied in vivo using preclinical animal models. However, this approach has limitations owing to species differences, which cannot be addressed using preclinical models. For instance, Sietsema et al. demonstrated significant differences in the absolute bioavailability of various drugs across species, highlighting the inability to predict bioavailability in humans^4^. Even between monkeys and humans, which share similarities in gastrointestinal physiology, there are substantial species differences in gut absorption^5^. As an alternative, cell models such as Caco-2 cells have been used to study intestinal absorption^6^. However, conventional in vitro systems have limitations that may hinder their utility. For example, the widely employed Caco-2 monolayer system has a lower expression of Phase I drug-metabolizing enzymes, including cytochrome P450 (CYP)3A4, CYP2C9, and CYP2C19^7,8^, compared to the human duodenum. Moreover, the expression of carboxylesterase (CES)1 in Caco-2 cells differs from that in the human small intestine^9^. Additionally, while Caco-2 cells express major transporters such as P-gp^10,11^, the expression patterns of other transporters, such as BCRP and multidrug resistance-associated proteins, differ considerably from those in human duodenal and jejunal tissues^7,12^. These limitations are critical for the study of the active transport and metabolism of orally administered drugs.

Given these challenges, microphysiological systems (MPS) have garnered attention as biomimetic systems that successfully model tissue units in terms of both structure and function^13,14^. MPS is expected to accurately recapitulate the key aspects of human organ structure and biological functional responses, offering opportunities to leverage them in drug discovery and development in the field of pharmaceutics. As for the intestine, there has been a surge in reports on gut MPS utilizing conventional cell models such as Caco-2 cells or human biopsy-derived materials seeded onto various MPS devices. For example, Shim et al. demonstrated improved CYP3A4 activity when Caco-2 cells were seeded on collagen-villi scaffolds within an MPS compared with conventional 2D culture^15^. Kasendra et al. developed gut MPS using biopsy-derived intestinal organoids, which recapitulated key anatomical and functional features of the small intestine, including 3D intestinal villi-like structures, multi-lineage differentiation, epithelial barrier function, enzymatic activity of brush border enzymes and mucus production^16^. However, the application of these gut MPS in the pharmaceutical field, particularly in absorption, distribution, metabolism, and elimination (ADME) sciences, is still limited, possibly because of the lack of comprehensive studies investigating the advantages of these emerging in vitro systems over conventional ones. With significant advances in MPS, there is great promise for their use in ADME sciences by enabling the direct observation of critical ADME parameters in humans, facilitating the selection of the best clinical candidates and predicting their disposition in humans, ultimately leading to the realization of precision medicine. Against this backdrop, the Japan Agency for Medical Research and Development (AMED) initiated an MPS project in Japan in 2017, aiming to generate an MPS that fully meets the end user’s needs and facilitates their application in drug discovery and development^17^. One of the projects, the ‘user-matching study’ seeks to establish a gut MPS that can readily be applied to ADME by addressing the prediction of gut absorption in humans using the MPS devices developed in the project.

In the present study, we established an MPS device called Fluid3D-X^®^, which is free of dimethylpolysiloxane, allowing for flow control within the system and enabling sequential sampling of media from the assay system. These features reflect the needs of end users for industrial applications. Human induced pluripotent stem cells (iPSCs)-derived small intestinal epithelial-like cells (hiSIECs), which possess gene expression profiles of ADME genes comparable to those of the adult intestine^18^, were seeded to establish gut MPS (gut MPS/Fluid3D-X). Using the developed gut MPS/Fluid3D-X, we examined the applicability of gut MPS for assessing the intestinal absorption of small-molecule drugs. This collaborative study between Daiichi Sankyo and AMED-MPS project was conducted as part of the ‘user-matching study.’

## Results

### Fabrication of Fluid3D-X device

**Fig. 1:**
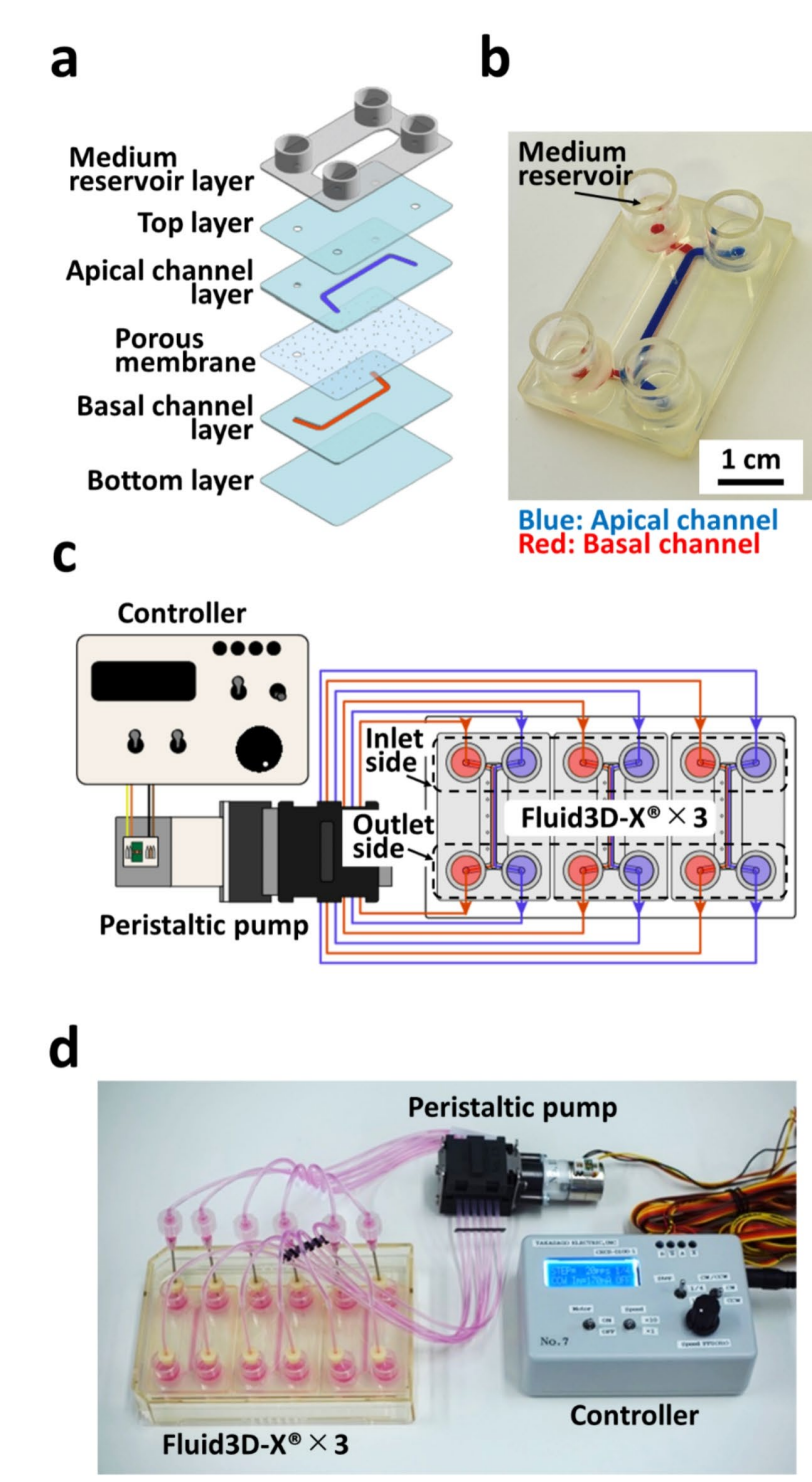
MPS hardware. MPS hardware. (a) Hard plastic film lamination fabricated using Fluid3D-X^®^. (b) Photograph of Fluid3D-X^®^. Blue: Apical channel; Red: Basal channel. (c) Illustration of Fluid3D-X^®^ and the medium perfusion setup. (d) Photograph of the medium perfusion setup.

Reflecting the needs of end users, we designed an MPS device in our previous study^19^ that was newly fabricated by Tokyo Ohka Kogyo for this study, namely Fluid3D-X^®^. As depicted in Fig. 1a, the device had a typical bilayered microchannel structure fabricated by the film lamination method with laser-processed films. The medium reservoirs were affixed around the microchannel ports (Fig. 1b). Culture media were perfused for cell culture using a peristaltic pump with tubes (Fig. 1c and d). The device enables the co-culture of cells across the membrane with continuous flow and takes a sufficient volume of samples from both influents and effluents in a sequential manner. More importantly, the device is made of polyethylene terephthalate (PET), free of PDMS, rendering the key features of limited adsorption of compounds to the device itself.

### Construction of gut MPS/Fluid3D-X for ADME study

**Fig. 2:**
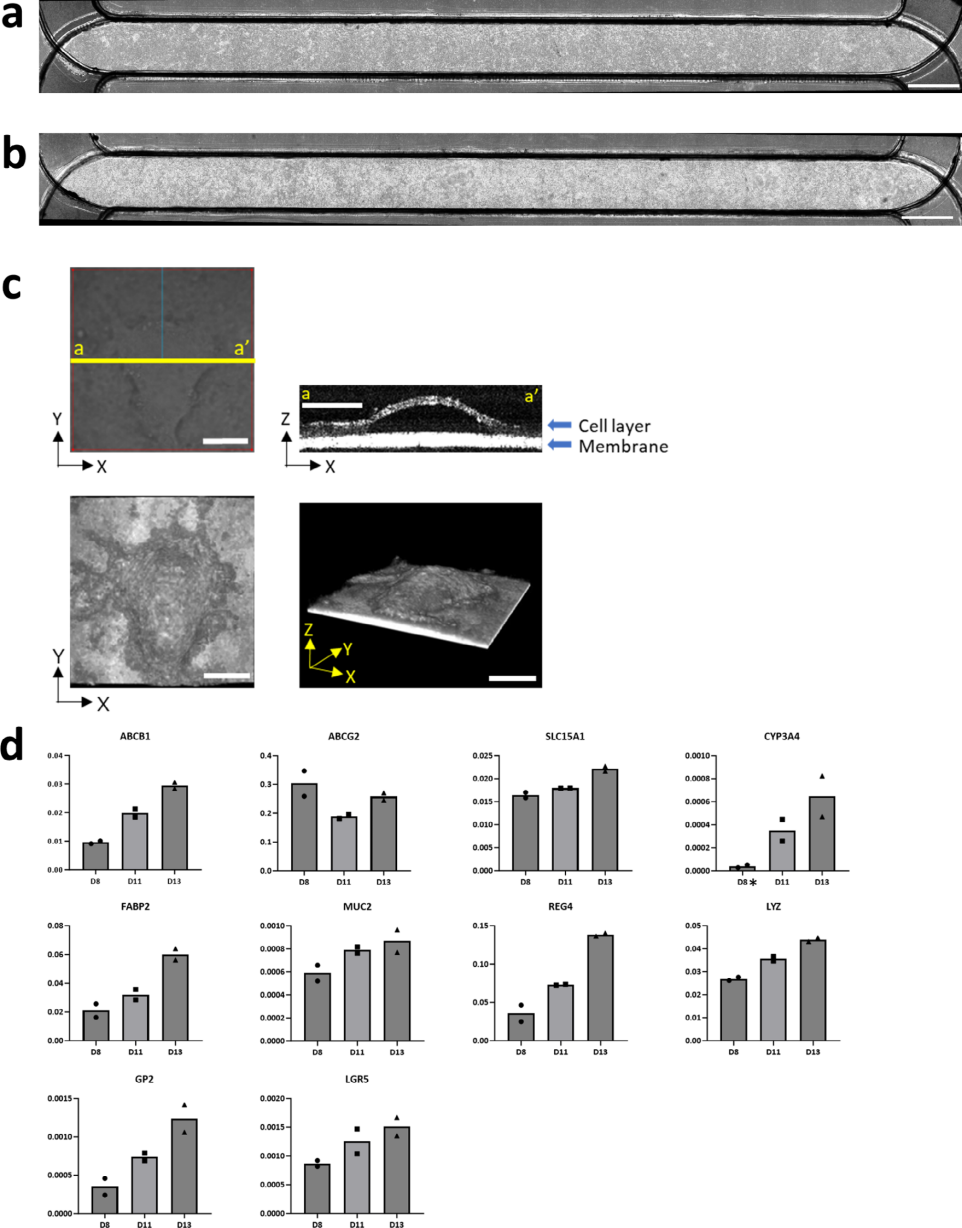
Construction of gut MPS/Fluid3D-X. Cells were cultured for 11 days, and phase-contrast images of gut MPS/Fluid3D-X were captured using an APX100 microscope (EVIDENT CORPORATION). Bright-field images of (a) liquid-liquid interface and (b) air-liquid interface cultures. Bar: 2 mm. (c) Cultured cells under ALI conditions (day 12) were observed using an optical coherence tomography (OCT) imaging system (CELL3 IMAGER ESTIER, SCREEN Holdings Co., LTD.). Top left: Bright field image of the observation area. Blue line indicates the scan direction. Top right: X-Z section image of yellow line (top left). The images were acquired at a 3 μm pitch across a 1 mm^2^ area. Bottom left: 3D dome structure obtained from the top. The image was generated utilizing Image J software (National Institutes of Health, Bethesda, MD, USA) Bottom right: 3D dome structure taken from the side. Scale bars: 250 μm. (d) Time-dependent expression of mRNAs (days 8, 11, and 13) was confirmed by probes that detect transporters, P450 enzymes, intestinal epithelial markers, and stem cell markers. The absolute value of each copy number was divided by that of EpCAM1. * Copy numbers were less than the lower limit of the calibration curve. Each bar represents the mean value (*n* = 2).

Gut MPS/Fluid3D-X was generated to assess the intestinal absorption of the small-molecule drugs, as shown in Fig. 2. hiSIECs were seeded on the porous membrane of Fluid3D-X^®^ and cultured under flow conditions for 11 days, either at the liquid-liquid interface (LLI; Fig. 2a) or the air–liquid interface (ALI; Fig. 2b). Gut MPS was successfully developed under both LLI and ALI conditions, as depicted in Fig. 2a and b. Based on previous reports by Shirai et al., who demonstrated improved expression and function of hiSIECs cultured under ALI conditions compared to LLI conditions^20^, we chose the gut MPS/Fluid3D-X model developed under ALI conditions for further characterization and transport studies. Morphological changes were observed over time after starting culture in flow under ALI conditions, as shown in Fig. 2c. A dome-shaped hiSIECs layer extruded from the porous membrane was observed from day 6 to the end of culture. The image of X-Z-section on day 12 suggested monolayer formations in dome-shaped hiSIECs layer (Fig. 2c, top right). Additionally, we characterized the mRNA expression levels of gut MPS/Fluid3D-X using quantitative PCR (qPCR), which revealed the maintenance and increased expression of drug transporters and drug-metabolizing enzymes (ABCB1, ABCG2, SLC15A1, and CYP3A4) over time (Fig. 2d). Importantly, the constructed gut MPS/Fluid3D-X exhibited FABP2, MUC2, REG4, LYZ, and GP2 expression, which are marker genes for epithelial cells, goblet cells, endocrine cells, Paneth cells, and M cells, respectively. This suggests that gut MPS/Fluid3D-X displays multilineage differentiation.

### Transport of P-gp and BCRP substrates across the hiSIECs monolayers in gut MPS/Fluid3D-X

**Fig. 3:**
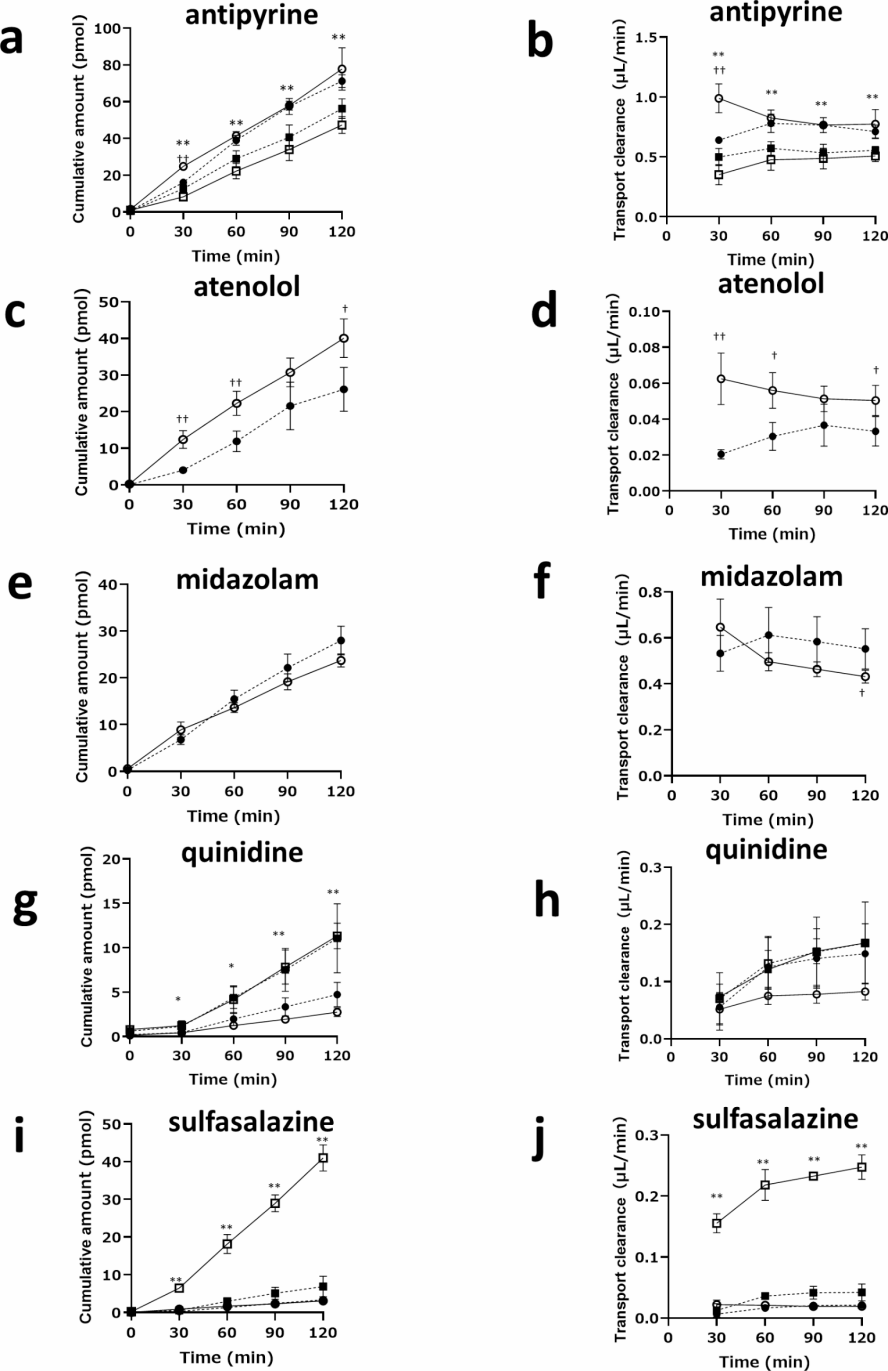
Assessment of gut transport across the hiSIECs monolayers in gut MPS/Fluid3D-X. Transport of antipyrine (a and b), atenolol (c and d), midazolam (e and f), quinidine (g and h), and sulfasalazine (i and j) across hiSIEC monolayers in gut MPS/Fluid3D-X. Cumulative amount of drugs and transport clearances as transported from apical to basal side in the presence (closed circle) or absence (open circle) of inhibitor cocktail after dosing of test substances to the apical side of gut MPS/Fluid3D-X. For antipyrine, quinidine, and sulfasalazine, transport from the basal to apical side was also examined in the presence (closed square) or absence (open square) of the inhibitor cocktail after dosing with antipyrine (a and b), quinidine (g and h), or sulfasalazine (i and j) to the basal side of the gut MPS/Fluid3D-X. †*p* < 0.05, ††*p* < 0.01; significant difference in apical-to-basal transport in the presence and absence of inhibitors. *p < 0.01, **p < 0.01; significant difference between the apical-to-basal transport compared to basal-to-apical transport in the absence of inhibitor. Each bar and error bar represent the mean value and SD, respectively (inhibitor(-): *n* = 4, inhibitor (+): *n* = 3).

Given that we observed leakage in the gut MPS/Fluid3D-X on day 13 of culture, as indicated by lucifer yellow permeability measurements (Supplemental Table S2), we conducted further transport experiments on day 11. At this time point, the permeability of lucifer yellow almost remained below 1.0 × 10^−6^ cm/s, indicating the presence of a leak-tight monolayer. Furthermore, the expression of drug transporters and drug-metabolizing enzymes was confirmed (Fig. 2d). In order to assess the functionality of P-gp and BCRP-mediated transport in the established gut MPS/Fluid3D-X, transport studies were conducted using quinidine and sulfasalazine, as typical P-gp and BCRP substrates, respectively. Quinidine or sulfasalazine was added to either the apical or basal side in the presence or absence of an inhibitor cocktail for CYP3A4, P-gp, and BCRP (ketoconazole 10 µM, PSC833 5 µM, and Ko143 5 µM). The cumulative amount of drugs and net clearance were calculated (Fig. 3g-j). Notably, the basal-to-apical transport was higher for both compounds than in the opposite direction, as observed in the cumulative amount and clearances with efflux ratios (ERs) of 2.02 and 12.7 for quinidine and sulfasalazine, respectively. Interestingly, directional transport diminished in the presence of transporter inhibitors, with a 45% decrease in quinidine intrinsic clearance and an 85% decrease in sulfasalazine. It is worth mentioning that we observed differences in the apical-to-basal transport between the two transcellular marker substances, antipyrine and atenolol, in terms of their sensitivity to inhibitors. While atenolol transport was sensitive to the inhibitors (Fig. 3c and d), antipyrine transport was not affected by the inhibitors (Fig. 3a and b). In addition, we observed apical-to-basal directional transport, specifically for antipyrine. Although these substances were selected as transcellular marker substances in the current study, our observations suggest that active transport processes contribute to a certain extent. Together, these findings provide compelling evidence for the functionality of P-gp and BCRP transport in gut MPS/Fluid3D-X.

### Metabolic conversion of CYP3A4 substrates in gut MPS/Fluid3D-X

**Fig. 4:**
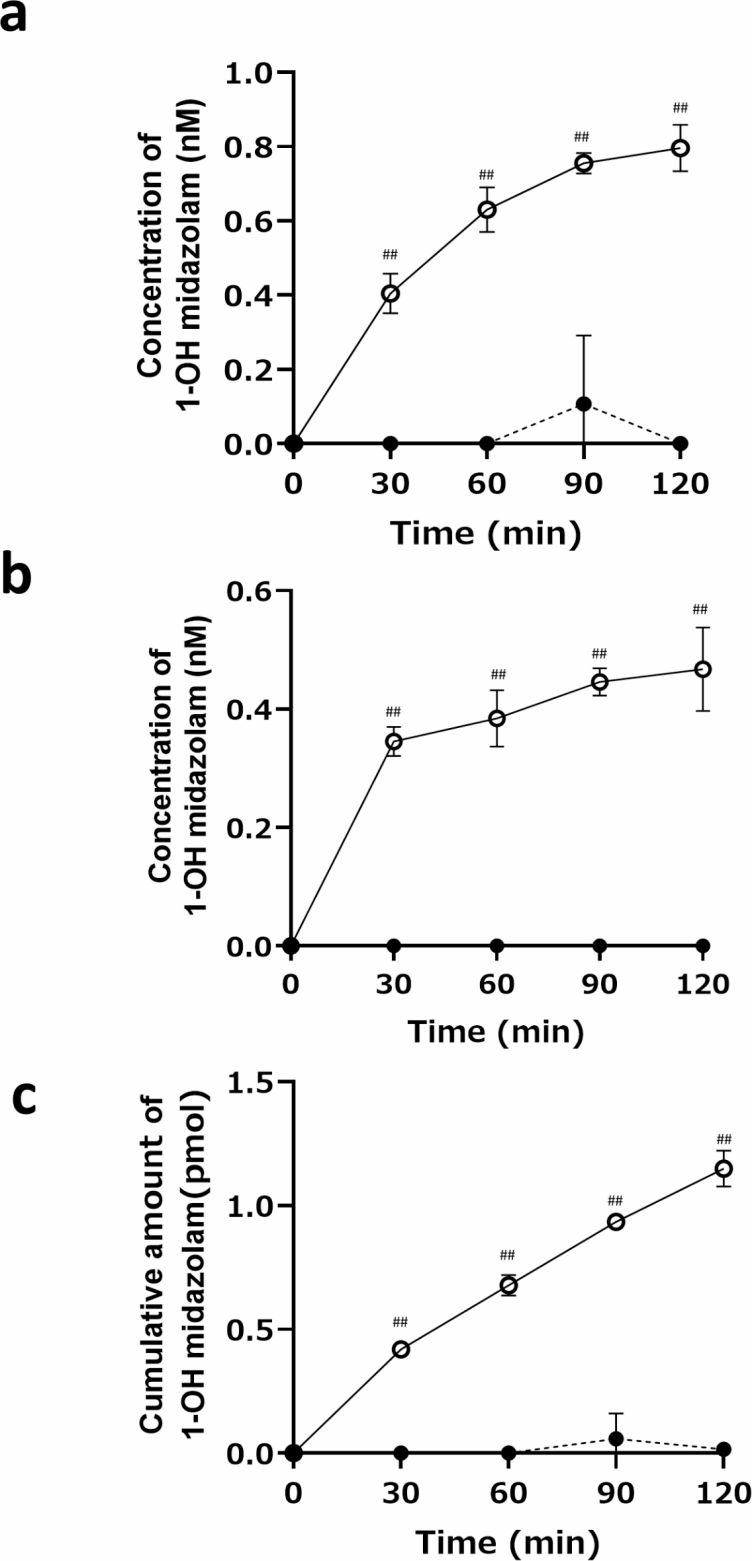
Metabolic activities of CYP3A4 in gut MPS/Fluid3D-X. Metabolism of midazolam in the gut MPS/Fluid3D-X. Midazolam was administered to apical side of gut MPS/Fluid3D-X in the presence (closed circles) or absence (open circles) of inhibitors, and concentration of its metabolite, 1-hydroxymidazolam was monitored in apical (a) or basal side (b). The total amount of 1-hydroxymidazolam formed by gut MPS/Fluid3D-X after dosing the apical side with midazolam is shown in Fig. 4c. ^##^*p* < 0.01; significant difference between the presence and absence of inhibitors. Each bar and error bar represent the mean value and SD, respectively (inhibitor(-): *n* = 4, inhibitor (+): *n* = 3).

The maintenance of metabolic activity in the gut MPS/Fluid3D-X was monitored using midazolam as a model substance. Similar to studies on transporter function, CYP3A4 activity was assessed by measuring the net transport from the apical side to the opposite side. Under these conditions, when CYP enzyme-mediated metabolism occurs, net transport should increase in the presence of CYP inhibitors, accompanied by a decrease in the formation of metabolites. Time-dependent transport of midazolam from the apical to basal side was observed (Fig. 3e and f), along with time-dependent formation of a metabolite (1-hydroxymidazolam) in both the apical and basal sides (Fig. 4), indicating the maintenance of metabolic activity. This was further confirmed by the effect of inhibitors, in which net fluxes increased along with decreased formation of 1-hydroxymidazolam. Together, these results suggest that metabolic activity is maintained in the gut MPS/Fluid3D-X.

## Discussion

With recent advances in bioengineering and materials engineering, there has been growing attention towards MPS as a system that closely mimics human physiology. Thus, MPS is a promising tool for drug discovery and development^14,21^. The field of ADME sciences can also benefit from MPS to address extrapolation of the preclinical ADME profiles of drugs to human^13,22,23^. We developed Fluid3D-X, which has a bilayered microchannel structure separated by a porous membrane and medium chambers in each port (Fig. 1), allowing the assessment of cell permeability and metabolism of drugs. F-hiSIEC™ is a commercially available human iPSCs-derived small intestinal epithelial-like cell. We selected these iPSCs-derived cells as model cells to minimize potential interlaboratory and interbatch variations. While iPSCs-derived cells may have the potential drawback of relative immaturity in terms of drug metabolism enzymes and transporter expression, F-hiSIEC™ possesses mRNA expression characteristics of intestine markers, drug-metabolizing enzymes, and drug transporters comparable to those of the adult small intestine^18^. Furthermore, the expression/activities of CYP3A4 were greatly improved by culturing F-hiSIEC™ under ALI conditions^20^. This motivated us to develop a gut MPS/Fluid3D-X under ALI conditions.

Cell adhesiveness and coverage of the filter by the cells were excellent in Fluid3D-X, providing an ideal platform for ADME studies under both LLI and ALI conditions for 11 d (Fig. 2). Interestingly, culturing under ALI conditions induced the formation of a dome-shaped structure, which has not been previously reported (Fig. 2c). However, the physiology of this structure requires further investigation. From a practical standpoint, the mRNA expression of ADME genes consistently increased over time, with the exception of ABCG2 and SLC15A1 (Fig. 2d). Furthermore, we observed the expression of marker genes, including FABP2, MUC2, REG4, LYZ, and GP2, which represent epithelial, goblet, endocrine, Paneth, and M cells, respectively. This indicates that gut MPS/Fluid3D-X exhibits multilineage differentiation. In particular, increased expression of LGR5 over time indicates the maintenance of stemness in the gut MPS/Fluid3D-X model.

Fluid3D-X offers two implementation methods for ADME studies: the “static method” where drugs are directly introduced to the donor side channel, and the “fluidic method” where drugs are continuously supplied from the donor chamber through flow. This is made possible by the larger channel volumes of Fluid3D-X (100 µl and 120 µl for the upper and lower channels, respectively) compared to the MPS developed by Emulate (28 µl in the top channel, 5 µl in the bottom channel). When channel volumes are small, the exchange of drugs across the cell monolayer can rapidly reach equilibrium, which hinders the accurate assessment of cell permeability in the “static method.” In our study on gut MPS/Fluid3D-X, we conducted experiments using the “static method” as a conventional approach. Barrier formation is typically monitored by measuring trans-epithelial cells electro resistance (TEER). However, to date, TEER measurements have not been implemented using Fluid3D-X. Instead, the integrity of the model was ensured by observation of lucifer yellow permeability, which measured below or nearly equal to 1.0 × 10^−6^ cm/s on the day of the transport experiments, indicating a leak-tight monolayer. We further utilized control substances with high and low permeability based on the effective permeability (P_eff_) in the human intestine, specifically antipyrine and atenolol, respectively, in our probe drug cocktail^24^. The P_app, apical to basal_ values of these compounds were measured at 20.1 and 1.31 × 10^−6^ cm/s for antipyrine and atenolol, respectively (Table 1). These findings support the notion that epithelial cells cultured under air-liquid interface (ALI) conditions form a barrier that allows for the evaluation of cell permeability. Furthermore, we successfully detected the activities of efflux transporters such as P-gp and BCRP in hiSIECs. Basal-to-apical directional transport of sulfasalazine and quinidine, typical substrates of transporters for BCRP and P-gp, respectively, was clearly observed. A significant decrease in directional transport by the inhibitors supported the functionality of BCRP and P-gp in the constructed MPS. Interestingly, we observed directional transport of antipyrine in the apical-to-basal direction, although the inhibitor cocktail had no effect. In addition, apical-to-basal transport of atenolol (Fig. 3c and d) was decreased by the inhibitor cocktails. It should be noted that inhibitor-sensitive apical-to-basal transport of atenolol was also observed in a different platform, gut MPS/Emulate (Supplemental Figure S1b). The unknown transporters induced by this culture condition may explain these findings.

**Table 1:** The summary Papp and ER calculated from transport study using gut MPS Data are presented as the mean ± SD (inhibitor (-): *n* = 4, inhibitor (+): *n* = 3).

	Antipyrine	Atenolol	Midazolam	Quinidine	Sulfasalazine
P_app, basal to apical_, inhibitor (-) (10^−6^ cm/s)	13.2 ± 1.1	not determined	not determined	4.37 ± 0.15	6.44 ± 0.45
P_app, apical to basal_, inhibitor (-) (10^−6^ cm/s)	20.1 ± 2.7	1.31 ± 0.19	11.2 ± 0.6	2.15 ± 0.33	0.505 ± 0.072
P_app, basal to apical_, inhibitor (+) (10^−6^ cm/s)	14.4 ± 0.7	not determined	not determined	4.37 ± 1.53	1.10 ± 0.29
P_app, apical to basal_, inhibitor (+) (10^−6^ cm/s)	18.5 ± 1.2	0.865 ± 0.173	14.4 ± 1.9	3.88 ± 1.12	0.557 ± 0.095
Efflux ratio (ER), inhibitor (-)	0.656 ± 0.120	-	-	2.02 ± 0.37	12.7 ± 2.3
Efflux ratio (ER), inhibitor (+)	0.780 ± 0.076	-	-	1.12 ± 0.63	1.97 ± 0.76

Regarding CYP3A4-mediated metabolism, which has remained an unsolved issue in in vitro gut models, we observed a net apical-to-basal transport of midazolam. This transport increased with the addition of inhibitors, reaching statistical significance based on cumulative data after 120 min (Fig. 3f). This increase was attributed to a decrease in the fraction metabolized in the gut due to the administration of a metabolism inhibitor, which is consistent with the time-dependent and inhibitor-sensitive formation of a metabolite (1-hydroxymidazolam) (Fig. 4). Notably, 1-hydroxymidazolam was preferentially secreted to the apical channel side, at twice the rate compared to the basal side, likely due to the difference in surface area exposed to the media. As a quantitative index, we calculated the fraction that escaped from the gut metabolism (Fg) value of midazolam to compare the estimated values from in vitro MPS studies with those from in vivo studies reported in the literature. The Fg value of midazolam was calculated to be 0.64, based on the ratio of apical-to-basal side transport clearance in the presence of an inhibitor to that in the absence of an inhibitor. This value was in good agreement with the clinical value (0.55^25^). Notably, the Fg value of midazolam obtained in this study was comparable to, or closer to, the in vivo value than that reported by Shirai et al. (0.74 ^20^). To determine whether Fg values obtained from in vitro models correlate with in vivo Fg in humans, the correlation between the in vitro and in vivo Fg values of multiple CYP3A4 substrates needs to be demonstrated, which may provide valuable insights into the use of gut MPS for the prediction of Fg.

In the present study, we also established gut MPS using the Emulate platform, where the same hiSIECs as those used in the gut MPS/Fluid3D-X studies were seeded (gut MPS/Emulate). Similar to gut MPS/Fluid3D-X, we confirmed the functionality of transporters (P-gp and BCRP) and metabolism (CYP3A4) using the same set of compounds and inhibitors as in the gut MPS/Fluid3D-X studies (Supplemental Figure S1). It should be noted that a direct comparison between the two assays (gut MPS/Fluid3D-X *versus *gut MPS/Emulate) in terms of evaluating gut absorption was not possible in this study, as the Fluid3D-X assays were conducted under static conditions after hiSIECs were cultured under flow, whereas the Emulate assays were performed under fluidic conditions during the transport assays. Regardless of the conditions, we demonstrated the potential of gut MPS in evaluating gut absorption kinetics in humans by confirming the functionality of transporters and P450 enzymes. In the gut MPS/Emulate, a notable decrease in the donor concentrations of midazolam was observed at the initial time points (Supplemental Figure S2). This reduction is likely attributed to sequestration by PDMS, an issue that has been documented in the context of reduced effective concentrations of MPS made of PDMS due to adsorption^26,27^. Similarly, the concentration of quinidine in the effluent decreased over time, with a reduction of 29% in the original drug solution within the first 6 h. However, this decrease was minimal at 24 h, at 82% of the corresponding inlet concentrations. This could be attributed to the continuous supply of drugs under fluid conditions. In the gut MPS/Fluid3D-X system, the donor concentrations were also lower than the measured concentrations of the drug solution; however, the concentrations remained relatively constant throughout the study (Supplemental Figure S3). These findings emphasize the importance of monitoring drug concentrations on the donor side for accurate calculation of P_app_ and Fg, regardless of the specific MPS used, as supported by a report by Carius et al.^27^. Although the decrease in donor concentrations was minimal and not a significant issue for the compounds tested in this study from a practical viewpoint, it is still crucial to ensure effective concentrations of test compounds. In addition to concerns regarding decreased effective drug concentrations, the same applies to substances that modulate cell function or differentiation. For example, the differentiation of hiSIECs is influenced by low-molecular-weight compounds such as forskolin^28^. Therefore, it is essential to validate the exposure concentrations of these supplements as well as investigational drugs to ensure proper cell function in the MPS devices for quality control.

In summary, we have demonstrated that the recently developed gut MPS model using a newly discovered MPS device can be applied to assess the intestinal absorption of small-molecule drugs. Gut MPS/Fluid3D-X recapitulates key aspects of gut physiology and allows for the assessment of absorption kinetics of drugs, including transporters and P450 enzyme substrates. Recent advances in MPS hold great promise for enabling the accurate prediction of disposition in humans. Our results suggest that the established platform can be used to assess the intestinal absorption of small-molecule drugs, aiding the selection of compounds with favorable absorption kinetics in humans for drug discovery and development processes.

## Methods

### Chemicals and reagents

Antipyrine, midazolam and ketoconazole and sulfasalazine-d4, were purchased from FUJIFILM Wako Pure Chemical Corporation. Sulfasalazine was from Tocris Bioscience. Quinidine was from Merck. Atenolol was from Combi-Blocks Inc. Antipyrine-d3 and quinidine-d3 were from Toronto Research Chemicals. 1-hydroxymidazolam and 4-hydroxymidazolam were obtained from Sigma-Aldrich. PSC833 was obtained from Angene Chemicals. Ko143 was obtained from ChemScene, LLC (additional information of the chemicals was described in Supplemental Table S3). All other chemicals used for the analyses were commercially available analytical-grade products.

### MPS hardware setup

Fluid3D-X^®^, a product of Tokyo Ohka Kogyo (Kanagawa, JAPAN), was used as a microfluidic chip for perfusion cell culture. Fluid3D-X^®^ has a typical bilayered microchannel structure separated by a porous membrane and medium chambers in each port. Fluid3D-X^®^ was fabricated by laminating polyethylene terephthalate (PET) films and porous membrane (pore size: 0.45 μm in diameter, 2000M12/640N453/A4, it4ip, Louvain-la-Neuve, Belgium) (Fig. 1a and b). To perfuse the culture medium, medium chambers on three Fluid3D-X^®^ in an ANSI/SLAS-compliant rectangular plate were connected to a peristaltic pump (AQ-RP6R-001, Takasago Fluidic Systems, Nagoya, JAPAN) with silicone tubes (CECS-C020-7, Takasago Fluidic Systems). Perfusion was performed at a flow rate of 3.8 ± 0.1 µL/min (This is the average value for the six channels of the pump when water was perfused under the same configuration) (Fig. 1c and d).

#### Cells

F-hiSIEC™ and culture media (seeding and maintenance media) were purchased from FUJIFILM Corporation (Lot No. VA2206-2, Tokyo, Japan).

### Establishment of gut MPS/Fluid3D-X

The seeding medium and culture medium of hiSIECs for LLI and ALI were obtained from FUJIFILM Corporation. hiSIECs were cultured according to the manufacturer’s instructions. Briefly, Fluid3D-X^®^ devices were coated with Corning Matrigel GFR Basement Membrane Matrix (Corning) on the day before seeding the cells. hiSIECs were seeded onto a Fluid3D-X^®^ device at 5.3 × 10^5^ cells/MPS and incubated for 5.5 h. The seeding medium was added to the device, and cell culture was started in flow for 11 days at 20 pps 1/4 (3.8 µL/min). After one day, the seeding medium was replaced with LLI culture medium, and the medium was changed every two or three days. ALI culture was performed using the same method as the LLI culture, but the apical channel medium was removed to expose the cells to air on day 4. On day 11 after seeding, the gut MPS was used for the assay.

### Gene expression of gut MPS/Fluid3D-X

Qiazol (Qiagen) was added to the ALI-cultured cells on the Gut MPS/Fluid3D-X on days 8, 11, and 13, and the cell lysates were collected. Total RNA was isolated from cell lysates using an RNAeasy mini kit (Qiagen), and cDNA was synthesized using the QuantiTect Reverse Transcription kit (Qiagen) according to the manufacturer’s protocol. Absolute quantification was performed using a Light Cycler 480 Probes Master (Roche) on a Light Cycler 480 System (Roche). The TaqMan probes used in this assay are summarized in Supplemental Table S1. The expression of EpCAM1 was employed as the intrinsic control.

### Transport studies of marker substrates of transporters and CYP3A4 and transcellular markers across the hiSIECs monolayers in gut MPS/Fluid3D-X

Eight gut chips were divided into two groups: with and without inhibitors. Preincubation with transport buffer containing HBSS (Thermo Fisher Scientific), 4.5 mg/mL glucose, and 10 mM MES (adjusted to pH 6.5, for the apical side) or 10 mM HEPES (adjusted to pH7.4, for the basolateral side) in the presence or absence of inhibitors (added to both sides) was performed for 10 min. Transport buffer containing drugs at designated concentrations (450 µL) was added to the apical (donor side) channel of gut MPS/Fluid3D-X, and deuterium-labeled compounds (antipiryne, quinidine, and sulfasalazine) containing buffer (450 µL) was added to the basolateral (acceptor side) channel of gut MPS/Fluid3D-X to initiate transport experiments. Concentration of drugs added to donor channel is as follows; antipyrine 1 µM, midazolam 1 µM, quinidine 0.5 µM, sulfasalazine 1 µM, atenolol 10 µM, d-antipyrine 1 µM, d-quinidine 0.5 µM and d-sulfasalazine 1 µM. Although we did not evaluate the cytotoxicity of the compounds used in the current study, the concentrations employed were significantly lower than the reported CC50 values^29–34^. This suggests that cytotoxic effects are unlikely to occur at the concentrations tested. Because all steps were performed under static conditions, 200 µL of buffer was transferred from the outlet to the inlet by a micropipette before collection of each sample. Serial sampling (150 µL) was performed at following time points (0, 30, 60, 90, and 120 min) from both the donor and acceptor sides. Equal volumes of fresh buffer were added after the sample collection. At the end of the experiment (120 min), the cell lysates were collected. The concentrations of drugs and/or metabolites in the transport buffer and cells were measured by LC-MS/MS. To observe the inhibitory effects of the inhibitor on compound transport, transport experiments were performed in the presence of an inhibitor cocktail containing ketoconazole (10 µM), PSC833 (5 µM), and Ko143 (5 µM) to inhibit CYP3A4, P-gp, and BCRP.

The permeability, efflux ratio, and Fg parameters were derived as follows:

The apparent permeability coefficient (Papp, cm/s) was calculated using Eq. (1):

1 $$\:Papp\:({10}^{-6}cm/s)\:={\frac{dQ}{dt}\times\:\frac{1}{MPSsurface\:area\:\times\:Co}}_{}$$

where dQ/dt, MPS surface area, and C_0_ represent the amount of the test compound transported to the receiver compartment per unit time when the linearity of its time-dependent transport was

apparently maintained, the culture area of the gut MPS/Fluid3D-X (0.64 cm^2^), and the

initial concentration of the test compound in the donor compartment.

2 $$\:Efflux\:ratio\:\left(ER\right)\:\:={\frac{Papp,\:basal\:to\:apical}{Papp,\:apical\:to\:basal}}_{}$$

where P_app, basal to apical_ and P_app, apical to basal_ represent the apparent permeability coefficient in the

basolateral-to-apical and apical-to-basolateral directions, respectively.

The intestinal availability (Fg) of CYP3A substrates was calculated based on a previous report^35^ using the following equation:

3 $$\:Fg\:\:={\frac{1}{2\frac{CLapical\:to\:basal\:(+inhibitor)}{CLapical\:to\:basal\:(-inhibitor)}-1}}_{}$$

where CLapical to basal (+ inhibitor) and CLapical to basal (-inhibitor) represent the transcellular transport clearance in the apical-to-basolateral direction in the presence or absence of the inhibitor, respectively.

### Quantification of transports of test articles by LC–MS/MS

The specimens were deproteinated with 3-times volumes of acetonitrile and centrifuged to obtain supernatants. For quantification of the drug concentration on the donor side, the specimens were further diluted 20 times with acetonitrile. Two microliters of each sample was subjected to LC–MS/MS analysis. An AB SCIEX QTRAP 5500 + mass spectrometer (Applied Biosystems, Foster City, CA) equipped with a Nexera X2 LC system (Shimadzu, Kyoto, Japan), operated in electron spray ionization mode, was used for the analysis. Chromatographic separation was performed at 40 °C on a GL Sciences Inc instrument. InertSustain C18 HP analytical column (2.1 I.D. × 100 mm, 3 μm) at a flow rate of 0.5 ml/min using the gradient method. The mobile phase consisted of 2 mmol/L ammonium acetate and 0.1% formic acid in water (mobile phase A) and 0.1% formic acid in acetonitrile (mobile phase B). Gradient condition (% of B concentration) 0.0–0.2 min, 0%; 0.2–0.25 min, 10%, 0.25–0.4 min, 25%, 0.4–2.5 min, 25%, 2.5–4.2 min, 95%; 4.2–4.21 min, 95 − 0%; 4.21–5.0 min, 0%. The ion spray voltage and temperature were set at 4500 V and 600 °C, respectively. The detailed LC–MS/MS conditions and information regarding the limits of qualification, concentration range, R² of the standard curve, and the range of differences between predicted and observed values summarized were summarized in Table 2, Supplemental Table S4 and S5.

**Table 2:** LC–MS/MS conditoins to analysis transports of test articles.

Compounds	Mass-to-charge	Retention time (min)	Ion mode	calibration curve range (nM)
midazolam	326.923→292.0	1.83	ESI positive	0.3–1000
1-hydroxymidazolam	342.01→168.0	1.89	ESI positive	0.3–1000
4-hydroxymidazolm	342.073→325.0	1.75	ESI positive	0.3–300
antipyrine	189.118→104.1	1.82	ESI positive	0.3–300
antipyrine-d3	192.075→106.0	1.82	ESI positive	1–300
quinidine	325.266→184.1	1.58	ESI positive	0.3–1000
quinidine-d3	328.113→163.0	1.58	ESI positive	0.3–1000
sulfasalazine	396.980→196.0	2.32	ESI positive	0.3–1000
sulfasalazine-d4	401.086→293.2	2.32	ESI negative	0.3–1000
atenolol	267.01→145.1	1.56	ESI positive	0.3–1000

### Statistical analysis

Statistical analyses were performed using GraphPad Prism 10. A 2-tailed Student’s t-test was employed to assess differences between the two groups (transport and/or metabolite formation in the presence and absence of the inhibitor). One-way ANOVA and Tukey–Kramer Multiple Comparisons tests were performed for more than two groups of observations. *P* value less than 0.05 was considered significant.

## Electronic supplementary material

Below is the link to the electronic supplementary material.

Supplementary Material 1

## Data Availability

The authors declare that the data supporting the findings of this study are available in the paper and its supplementary information files.
