# Supplementary material for: Development of a novel gut microphysiological system that facilitates assessment of drug absorption kinetics in gut
Source: Sci Rep. 2024 Dec 2;14:29921. doi: 10.1038/s41598-024-80946-6 (PMC11612460; doi:10.1038/s41598-024-80946-6)
Supplement: Supplementary file 1 — Supplementary Material 1 [file 41598_2024_80946_MOESM1_ESM.docx]

**Supplementary Materials Table of Contents**

Materials and Methods

Figure S1. Assessment of gut absorption in gut MPS using Emulate MPS platform

Figure S2. Time-donor concentration profiles of test articles for transport experiments using Emulate MPS platform

Figure S3. Time-donor concentration profiles of test articles for transport experiments using gut MPS/Fluid3D-X

Table S1. Taq man probes for quantitative PCR

Table S2. Transport of lucifer yellow across gut MPS/Fluid3D-X monolayers after culturing hiSIECs over time

Table S3. Chemicals and reagents details

Table S4. Standard curves for the quantification using LC-MS/MS

Table S5. Standard curves for the quantification using LC-MS/MS for Supplementary Figure S2 and S3

**Supplementary Materials:**

**Materials and Methods.**

**Culture of F-hiSIEC on Emulate chips**

After UV activation, according to the manufacturer’s protocol, the chips (Emulate, #10231-2 Lot 000904) were coated with 5% Matrigel solution (Corning, #354230). F-hiSIEC was thawed and suspended in seeding medium according to the manufacture’s protocol. The suspension of F-hiSIEC (6.0×10^6^ cells/mL, 35-40 μL/chip) was transferred to the chip and incubated at 37ºC overnight. The cells were then cultured in maintenance medium under perfusion culture conditions at a flow rate of 15 μL/hr from day1 to day 4 and 30 μL/h from day 5 to day9. Gravity wash and prime cycle were performed when necessary. Additionally, the membrane strain rate was set to 2% at a frequency of 0.15 Hz from day 3 to day 5 and to 10% from day 6 to day 9. The permeability of the test drugs was assessed on day 9.

**Assessment of permeability of test drugs using gut MPS/Emulate**

The test drugs were added to the culture medium. The top and bottom channels of the chip were perfused with the media at a flow rate of 30 μL/h. Top channel cocktail: 1 μM antipyrine, 1μM midazolam, 0.5μM quinidine, 1μM sulfasalazine, and 10μM atenolol; bottom channel cocktail: 1 μM antipyrine-d3, 0.5μM quinidine-d3, and 1μM sulfasalazine-d4. Inhibitors (10μM Ketoconazole, 5μM PSC833 and 5μM Ko143) were added as a cocktail to both the channels. Media were collected from the outlet chamber at 6, 24, 30, and 48 h.

Transport clearance (PS) across the cell monolayer of the test drugs was calculated as follows:

$PS ={\frac{Cumulative amount in the acceptor channel}{Area under the concentration in the donor channel}}$ (1)

The metabolic clearance (CLmet) of midazolam was calculated as follows.

$CLmet={\frac{Sum of cumulative amount of metabolite (1'-hydroxymidazolam or 4-hydroxymidazolam) in the donor and acceptor channels}{Area under the concentration of midazolam in the donor channel}}$ (2)

**Assessment of permeability of lucifer yellow using gut MPS/Fluid3D-X**

F-hiSIECs were cultured on four or two chips for a duration of 11-13 days. For permeability assay, chips were preincubated with transport buffer containing HBSS (Thermo Fisher Scientific, #14025-092), 4.5 mg/mL glucose, and 10 mM HEPES (adjusted to pH7.4) for 1 hr. Following the removal of the buffer from each reservoir, 300 µM lucifer yellow (Fuji Film, # 125-06281) containing the transport buffer was introduced to the inlet of the apical channel, while the transport buffer was added to the inlet of the basal channel. Samples (50 µL) were immediately collected from each channel, and the chips were subsequently incubated under static conditions for 30 minutes. After thorough mixing via pipette manipulation, samples were collected and the concentration of lucifer yellow was quantified using a fluorescent microplate reader (Cytation 5, Agilent).


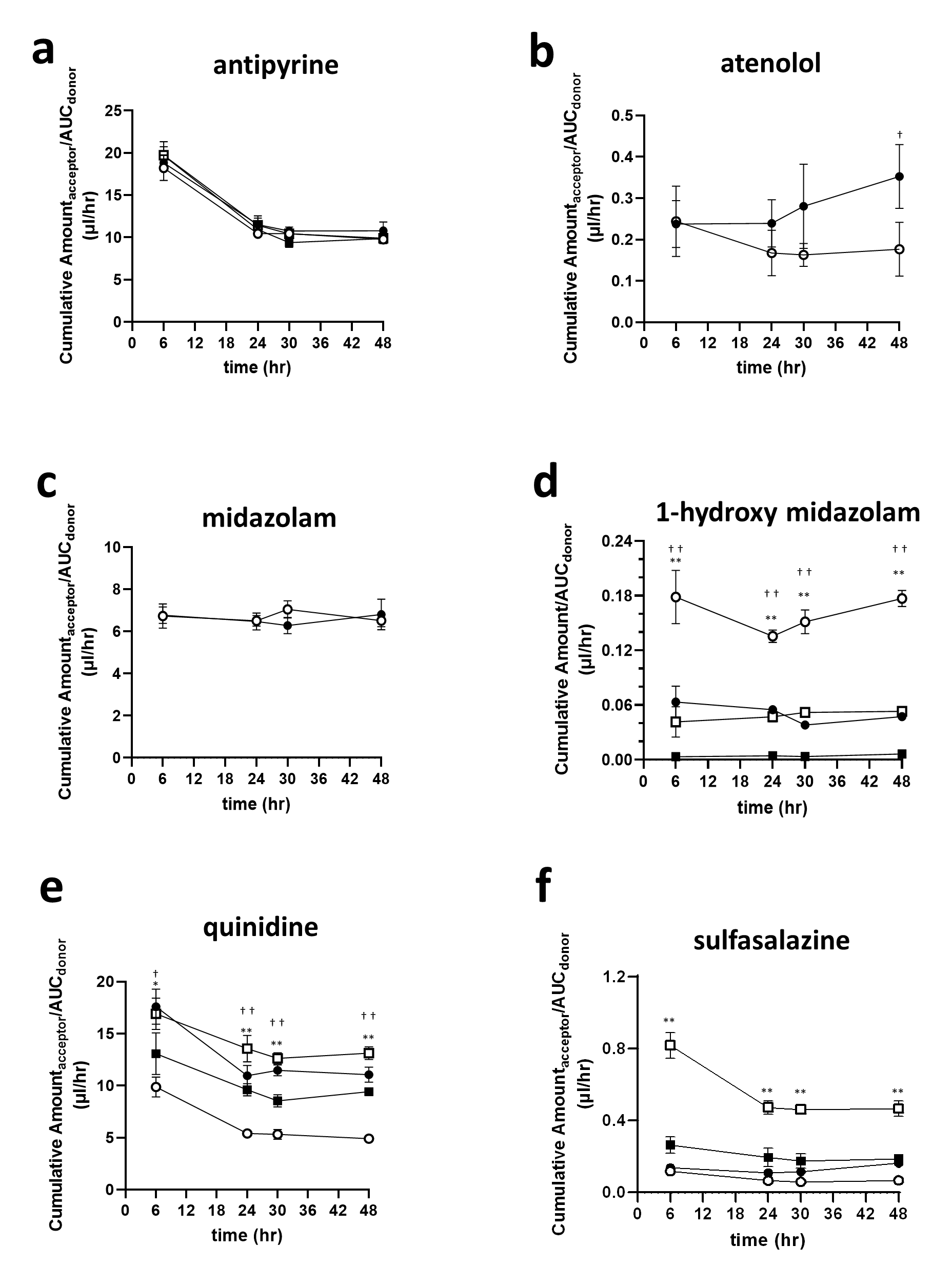
**Figure S1. Assessment of gut absorption in gut MPS using Emulate MPS platform**

Transport of antipyrine (a), atenolol (b), midazolam (c), 1-hydroxymidazolam (d), quinidine (e), and sulfasalazine (f) across the hiSIECs monolayers in gut MPS/Emulate. Antipyrine, atenolol, midazolam, quinidine and sulfasalazine were administered to apical channel of gut MPS/Emulate in the presence (closed circle) or absence (open circle) of inhibitor cocktail after

and the effluents from the basal channel were collected. For antipyrine, quinidine, and sulfasalazine, transport from the basal-to-apical channel was also examined in the presence (closed square) or absence (open square) of the inhibitor cocktail after dosing those drugs to the basal side of the gut MPS/Emulate. Data are shown as transported clearance as calculated by the cumulative amount of drugs transported divided by the AUC of the donor, as described in the Materials and Methods section. As for midazolam, after administration of midazolam to the apical channel of gut MPS/Emulate in the presence (closed symbols) or absence (open symbols) of inhibitors, formation of a metabolite, 1-hydroxymidazolam, was observed in the apical side (circles) or basal side (squares). Data represent the mean ± SD.

^†^p < 0.05, ^††^p < 0.01; significant difference in apical-to-basal transport in the presence and absence of inhibitors. ^*^p < 0.01, ^**^p < 0.01; significant difference between the apical-to-basal transport compared with basal-to-apical transport in the absence of inhibitor.

**
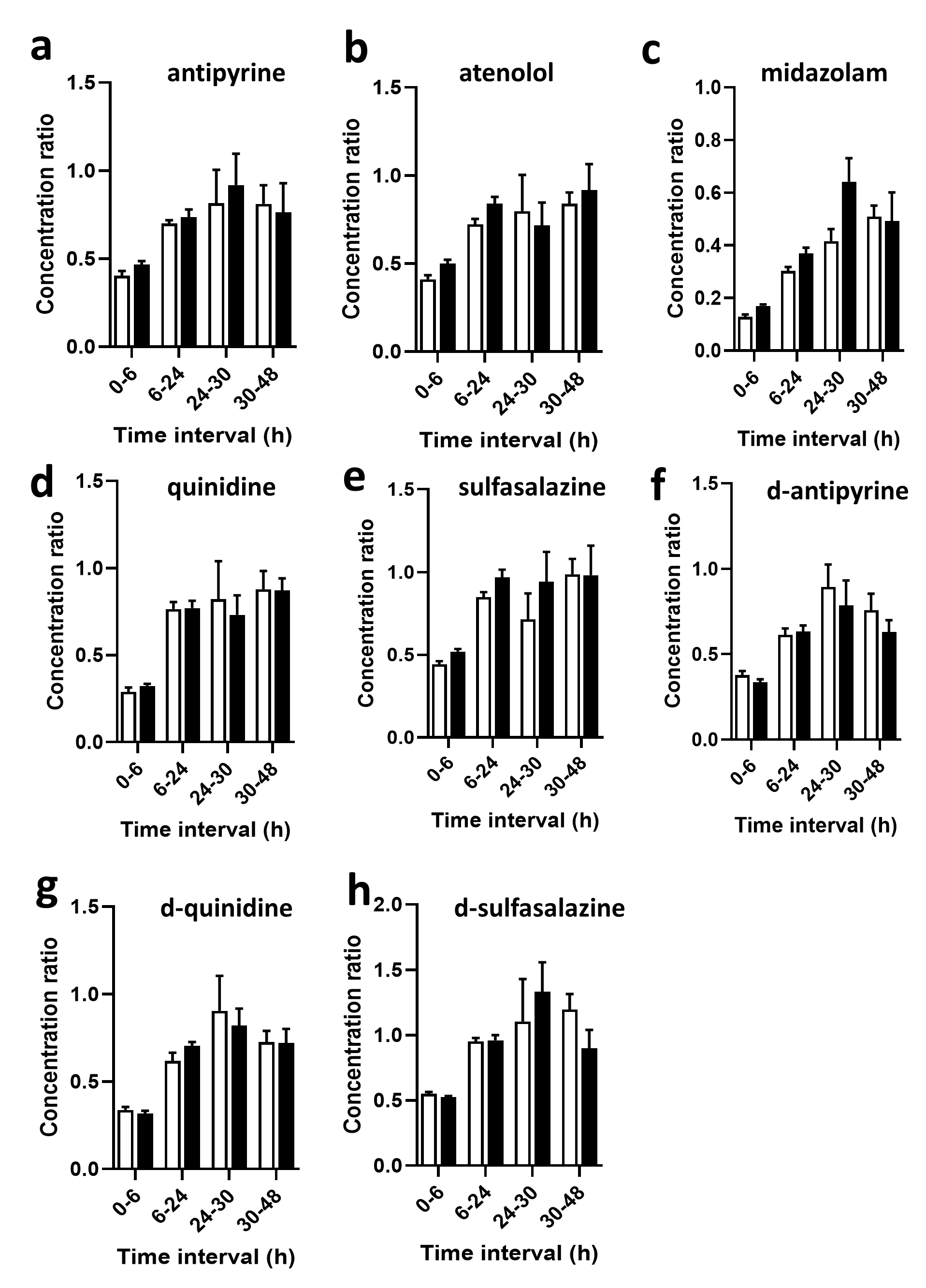
**

**Figure S2. Time-donor concentration profiles of test articles for transport experiments using Emulate MPS platform**

Time-donor concentration profiles of antipyrine (a), atenolol (b), midazolam (c), quinidine (d), sulfasalazine (e), d-antipyrine (f), d-quinidine (g), and d-sulfasalazine (f). Antipyrine (a), atenolol (b), midazolam (c), quinidine (d), and sulfasalazine (e) were infused into the apical channel of the gut MPS/Emulate in the presence (closed column) or absence (open column) of inhibitor cocktails, and effluents from the apical channel were sequentially collected and drug concentrations were measured. Donor concentrations were presented as the ratio of the measured concentration of effluents at each time point to the measured concentration of the original drug solution. For d-antipyrine (f), d-quinidine (g) and d-sulfasalazine (f), drugs were infused into basal channel of gut MPS/Emulate in the presence (closed column) or absence (open column) of inhibitors cocktails and effluents from the basal channel were sequentially collected and drug concentrations were measured. Approximately 6 h after starting the perfusion of drugs, drug concentrations reached a plateau. Notably, the donor concentration of midazolam was almost half of the spiked concentration, presumably due to the adsorption of the devices.

* Indicates a significant difference between the presence and absence of inhibitors (P < 0.05).

**
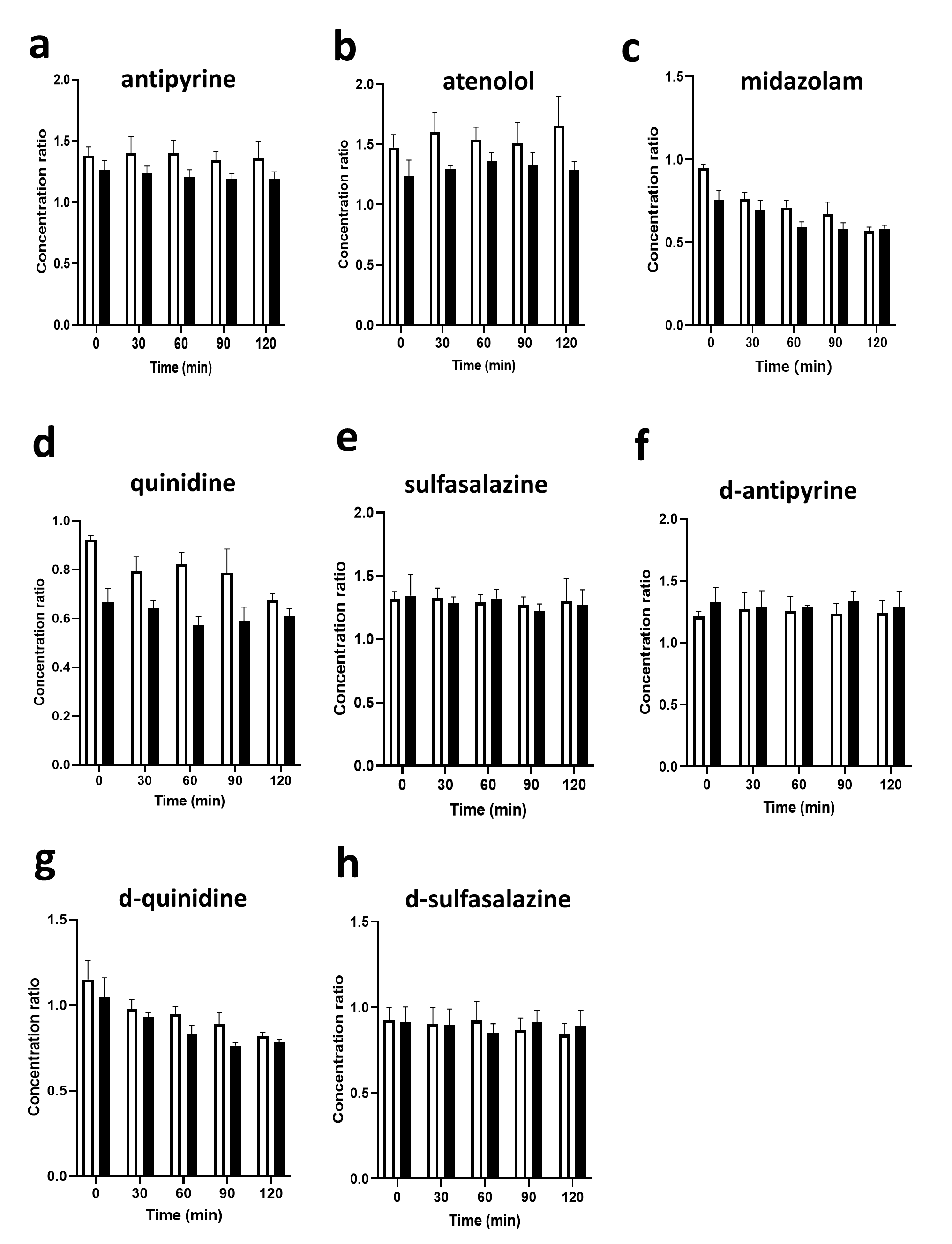
**

**Figure S3. Time-donor concentration profiles of test articles for transport experiments using gut MPS/Fluid3D-X**

Time-donor concentration profiles of antipyrine (a), atenolol (b), midazolam (c), quinidine (d), sulfasalazine (e), d-antipyrine (f), d-quinidine (g) and d-sulfasalazine (f). Antipyrine (a), atenolol (b), midazolam (c), quinidine (d), and sulfasalazine (e) were added to the apical channel of the gut MPS/Fluid3D-X in the presence (closed column) or absence (open column) of inhibitor cocktails, and serial sampling (150 µL) was performed at the following time points (0, 30, 60, 90, and 120 min) from both the donor side and drug concentrations were measured. Donor concentrations are presented as the ratio of the measured concentration of the apical channel at each time point to the measured concentration of the original drug solution. Notably, the concentrations remained relatively constant throughout the study period.

### Table S1. Taq man probes for quantitative PCR

|  | Gene name | Catalog No. |
| --- | --- | --- |
| 1 | GAPDH | Hs99999905_m1 |
| 2 | EpCAM | Hs00901885_m1 |
| 3 | HPRT1 | Hs99999909_m1 |
| 4 | FABP2 | Hs01573164_g1 |
| 5 | MUC2 | Hs00159374_m1 |
| 6 | REG4 | Hs00230746_m1 |
| 7 | LYZ | Hs00426232_m1 |
| 8 | GP2 | Hs00426805_m1 |
| 9 | CYP3A4 | Hs00604506_m1 |
| 10 | ABCB1 | Hs00184500_m1 |
| 11 | ABCG2 | Hs01053790_m1 |
| 12 | SLC15A1 | Hs00192639_m1 |

**Table S2. Transport of lucifer yellow across gut MPS/Fluid3D-X monolayers after culturing hiSIECs over time**

| Culture days | Papp, apical to basal, (10^-6^ cm/s) | | | | | |
| --- | --- | --- | --- | --- | --- | --- |
|  | C-1 | C-2 | C-3 | C-4 | average | SD |
| 11 | 0.537 | 0.730 | 0.275 | 1.16 | 0.675 | 0.322 |
| 12 | 0.834 | 1.38 | 0.437 | 0.703 | 0.839 | 0.345 |
| 13 | 1.17 | 10.9 |  |  | 6.03 |  |

F-hiSIECs were cultured on four or two chips for a duration of 11-13 days. The permeability of lucifer yellow was evaluated to assess membrane integrity. Data for the three types of culture days were obtained independently. Data are presented as the mean ± SD for day 11 and 12 samples (n=4) and mean ± SD for day 13 samples (n=2).

**Table S3. Chemicals and reagents details**

| Chemical  name | manufacturer | Cat.No | Grade | CAS No. | Purity | Stock sol.  (mM) |
| --- | --- | --- | --- | --- | --- | --- |
| Antipyrine | FUJIFILM Wako Pure Chemical Corporation | 018-11112 | for Biochemistry | 60-80-0 | 99.0+% (After Drying)(Titration) | 10 |
| Midazolam | FUJIFILM Wako Pure Chemical Corporation | 135-13791 | for Biochemistry | 59467-70-8 | 97.0+% (Titration) | 10 |
| Ketoconazole | FUJIFILM Wako Pure Chemical Corporation | 116-00551 | for Biochemistry | 65277-42-1 | 98.0+% (HPLC) | 100 |
| Sulfasalazine-d4 | TRC-S699089-2.5MG | S699089 | TRC, Research Materials | 1346606-50-5 |  | 10 |
| Sulfasalazine | Tocris Bioscience | 4935 |  | 599-79-1 | ≥97% (HPLC) | 10 |
| Quinidine | Sigma-Aldrich | 22600-10G-F | 100 | 56-54-2 | ≥98.0% (dried material, NT) | 10 |
| Atenolol | Combi-Bloks | NA-0037 |  | 29122-68-7 | 98% | 10 |
| Antipyrine-d3 | TRC-A697502-10MG | A697502 | TRC, Research Materials | 65566-62-3 |  | 10 |
| Quinidine-d3 | TRC-Q685002-2.5MG | Q685002 | TRC, Research Materials | 1267657-68-0 |  | 10 |
| 1-hydroxymidazolam | Cayman Chemical | 10385 |  | 59468-90-5 | ≥95% | 1 |
| 4-hydroxymidazolam | Sigma-Aldrich | UC431-5MG |  | 59468-85-8 | ≥ 97 % | 1 |
| PSC833(Valspodar) | Angene | AG0015BN |  | 121584-18-7 | 99% | 10 |
| Ko143 | ChemScene | CS-0298 |  | 461054-93-3 | 99.83% | 50 |
| Lucifer yellow | FUJIFILM Wako Pure Chemical Corporation | 125-06281 | for Biochemistry | 67769-47-5 |  | 100 |

**Table S4. Standard curves for the quantification using LC-MS/MS**

| **Chemical name** | **Concentration range (nM)** | ***R^2^*** | **LOQ (nM)** | **100-Accurary (%)** |
| --- | --- | --- | --- | --- |
| Atenolol (ATL) | 0.3, 1, 3, 10, 30, 100, 300, 1000 | 0.983271 | 0.3 | 12 ^a^ (4.0 ~ 54) |
| Antipyrine (ATP) | 0.3, 1, 3, 10, 30, 100, 300 | 0.991418 | 0.3 | 6 ^a^ (1.0~ 31.1) |
| Antipyrine-*d*3 (dATP) | 1, 3, 10, 30, 100, 300 | 0.988235 | 1 | 9 ^a^ (2.0 ~ 26.6) |
| Midazolam (MD) | 0.3, 1, 3, 10, 30, 100, 300, 1000 | 0.980892 | 0.3 | 8.5 ^a^ (2.0 ~ 23.5) |
| 1'-Hydroxymidazolam  (1HMD) | 0.3, 1, 3, 10, 30, 100, 300, 1000 | 0.99361 | 0.3 | 5 ^a^ (2 ~ 46.6) |
| 4-Hydroxymidazolam  (4HMD) | 0.3, 1, 3, 10, 30, 100, 300, 1000 | 0.990224 | 0.3 | 8.8 ^a^ (0 ~ 27.5) |
| Quinidine | 0.3, 1, 3, 10, 30, 100, 300, 1000 | 0.980298 | 0.3 | 3.2 ^a^ (0.2 ~ 25.6) |
| Quinidine-*d*3 | 0.3, 1, 3, 10, 30, 100, 300, 1000 | 0.982477 | 0.3 | 8.9 ^a^ (1.4 ~ 20) |
| Sulfasalazine (SFL) | 0.3, 1, 3, 10, 30, 100, 300, 1000 | 0.995405 | 0.3 | 4.8 ^a^ (0 ~ 11) |
| Sulfasalazine-*d*4 (dSFL) | 0.3, 1, 3, 10, 30, 100, 300, 1000 | 0.99102 | 0.3 | 6.8 ^a^ (2.0 ~ 17) |

^a^ median; values in the parenthesis represent the range.

**Table S5. Standard curves for the quantification using LC-MS/MS for Supplementary Figure S2 and S3**

| **Chemical name** | **Concentration range (nM)** | ***R*^2^** | **LOQ (nM)** | **100-Accurary (%)** |
| --- | --- | --- | --- | --- |
| Atenolol (ATL) | 0.3, 1, 3, 10, 30, 100, 300, 1000 | 0.98903 | 0.3 | 5.2 (2 ~ 16) |
| Antipyrine (ATP) | 0.3, 1, 3, 10, 30, 100, 300 | 0.98744 | 0.3 | 9.9 (2.6 ~ 34.3) |
| Antipyrine-*d*3 (dATP) | 1, 3, 10, 30, 100, 300 | 0.990025 | 1.0 | 5.25 (2.0 ~ 14.4) |
| Midazolam (MD) | 0.3, 3, 10, 30, 100, 300, 1000 | 0.981685 | 0.3 | 13.3 (0.5 ~ 19.3) |
| 1'-Hydroxymidazolam (1HMD) | 0.3, 1, 3, 10, 30, 100, 300, 1000 | 0.982676 | 0.3 | 2.25 (0 ~ 25.7) |
| 4-Hydroxymidazolam (4HMD) | 0.3, 1, 3, 10, 30, 100, 300 | 0.993212 | 0.3 | 6.5 (1.0 ~ 12) |
| Quinidine | 0.3, 1, 3, 10, 30, 100, 300, 1000 | 0.988831 | 0.3 | 9 (0.3 ~ 17.4) |
| Quinidine-d3 | 0.3, 1, 3, 10, 30, 100, 300, 1000 | 0.989428 | 0.3 | 7.5 (0.1 ~ 14.3) |
| Sulfasalazine (SFL) | 0.3, 1, 10, 30, 100, 300, 1000 | 0.985056 | 0.3 | 12.7 (2.1 ~ 29.9) |
| Sulfasalazine-d4(dSFL) | 0.3, 1, 3, 10, 30, 100, 300, 1000 | 0.991618 | 0.3 | 5.5 (3.0 ~ 16) |

^a^ median; values in the parenthesis represent the range.
